# The Gritti-Stokes Amputation: Is It Still a Reliable Technique in the 21st Century? A Narrative Review

**DOI:** 10.3390/medicina60060911

**Published:** 2024-05-30

**Authors:** Marwan Garaud, Louis-Romée Le Nail, Bandar Hetaimish, Julien Berhouet, Ramy Samargandi

**Affiliations:** 1 Service de Chirurgie Orthopédique et Traumatologique, Clinique Jeanne d’Arc, 45500 Gien, France; 2 Service de Chirurgie Orthopédique et Traumatologique, Centre Hospitalier Régional Universitaire (CHRU) de Tours, 1C Avenue de la République, 37170 Chambray-les-Tours, France; lr.lenail@chu-tours.fr (L.-R.L.N.); julien.berhouet@gmail.com (J.B.); ramy.samargandi@hotmail.com (R.S.); 3 Department of Orthopedic Surgery, Faculty of Medicine, University of Jeddah, Jeddah 23218, Saudi Arabia; bmhetaimish@uj.edu.sa

**Keywords:** Gritti–Stokes, amputation, below-knee amputation, transfemoral amputation, rehabilitation, prosthetic fitting, peripheral vascular disease

## Abstract

Lower limb amputation is a common surgical procedure performed worldwide. Many individuals require amputation due to various circumstances, with amputations occurring above or below the knee. Surgeons rely on published research to determine the most appropriate technique based on intraoperative and postoperative outcomes. The Gritti–Stokes amputation (GSA) procedure, introduced in 1857, has shown positive results in terms of primary wound healing, reduced mortality rates during and after surgery, and accelerated healing and mobility. However, due to the need for highly trained surgeons and limitations in functional and cosmetic prosthesis fitting, concerns have been raised regarding its utility. Additionally, the procedure is underutilized in cases where it could potentially yield better results. This article provides a comprehensive review of the documented benefits of GSA, suitable candidate selection, limitations, various modifications, and a comparison with traditional approaches to lower limb amputation. The review is focused on evidence published in the last 100 years.

## 1. Introduction

In the United States, about 2 million people suffer from limb loss, with an estimated 185,000 amputations yearly [1]. In France, from 2011 to 2020, a total of 116,866 major lower limb amputations were recorded [2]. The classification of limb loss includes major limb loss and minor limb loss. Major limb loss is often trans-femoral, through-knee amputation, or trans-tibial amputation. A minor limb loss is an amputation of the toes or mid-foot [3]. Above-knee amputation (AKA) is usually indicated in patients with peripheral vascular disease (PVD), significant trauma, malignant tumors, resistant incurable bone and prosthetic joint infection, and congenital defects. Below-knee amputations (BKA) are mainly for more distal injuries. An AKA requires more energy than a BKA [3,4]. Amputations occur in all age groups but are more common in those over 65 years old, particularly among individuals with diabetes, vascular disorders, and other medical comorbidities [1,5].

The residual problem of AKA has been accepted throughout time. The major purpose of the procedure is to keep the femur in the center location of the skin envelope. The loss of hamstring and hip adductor muscles prevents hip adduction and extension. The short stump is directly related to abductor deformity when not undergoing adductor myodesis, where sometimes adductor insertion is attached to prevent this issue [6].

A through-knee amputation, also known as knee disarticulation, keeps the quadriceps attached to the patella. This prevents unopposed abductors, as observed in the case of not performing adductor myodesis, as seen in the case of the standard transfemoral amputation. The stump’s end is made of tissue suited for kneeling. The hip muscles have a long lever arm that acts in the physiological field to control forces. This type of amputation gives more comfort, proprioception, and stability than the ischial weight-bearing AKA prosthesis [7]. Although through-knee amputation theoretically improves mobility, it is not routinely performed in most centers as it may result in a high complication rate due to the long flaps of tissue close to the wound over the femoral condyles. A bulbous stump is often difficult to fit with a prosthesis [8].

After World War II, the technique lost favor due to prosthetic considerations. To implant the stump in more elegant prosthetic support, Mazet and Hennessy described a new technique for through-knee amputation to overcome the bulky distal stump by resecting the medial, lateral, and posterior surface of femoral condyles by excising the patella to make a conical distal stump which improved prosthetic fitting. This technique requires a smaller skin flap, which may decrease wound complications [9]. This procedure has recently regained popularity [10]. Callander described another technique for lower third femur amputation in 1936, claiming that this was superior to the older Gritti–Stokes technique due to the anterior flaps utilizing the soft tissues of the upper leg up to the level of the tibial tuberosity, as well as other modifications such as removing the patella and using fewer sutures with better cosmetic results [11]. Such claims have been made by other authors as well.

**Figure 1:**
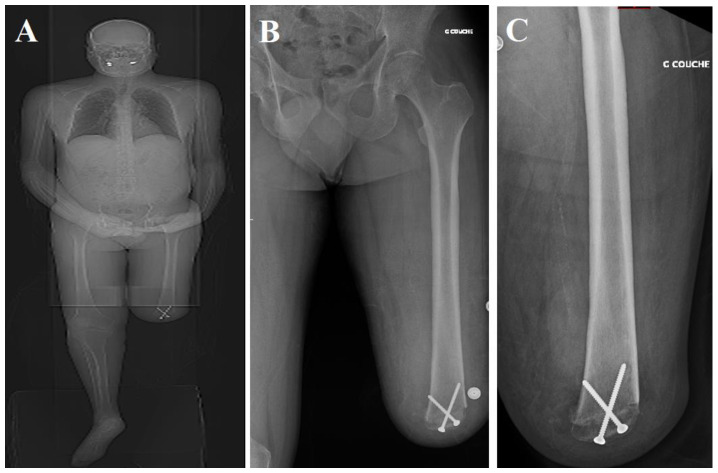
Radiological imaging demonstrating GSA: (**A**) long-standing film, (**B**) anteroposterior view of left femur, and (**C**) lateral view.

Our interest here is in the technique for the through-knee amputation that is known as Gritti–Stokes amputation (GSA) or osteoplastic amputation at the distal end of the femur [12] (Figure 1). The GSA technique, like Mazet and Hennessy’s, attempted to overcome the inadequacies of through-knee amputation but fell out of favor in the late 1950s and early 1960s for the same reasons as cited above [9]. The authors have decided to undertake a narrative review of the GSA, gauge its benefits, its historical perspective, and its use over time, and, based on the evidence thus reviewed, advocate for reviving its popularity. Evidence has been collected after going through various studies and reviews of original articles that either recommended or criticized GSA.

## 2. Materials and Methods

We searched PubMed and Embase for “Gritti Stokes Amputation” or “Gritti Stokes” between 1900 and 2023. We only included original papers, excluding short correspondences, notes, abstracts, case reports, and theses. All identified publications in the literature were reviewed and meticulously evaluated.

## 3. Historical Perspective

François Chopart’s description of the amputation procedure for the ankle joints in 1791 inspired contemporary and future surgeons to apply the approach to enhance amputation outcomes through the knee joint, including Smith’s first amputation of the knee joint performed in 1825, followed by improvements over the years [9,13,14]. Subsequently, in 1857, Rocco Gritti, a young surgeon at the Ospedale Maggiore in Milan, published an article giving a short history of amputations and described a method of amputation through the knee, using the patella as an osteoplastic flap to provide an end-bearing stump. Gritti modified Smith’s technique by using the patella as a weight-bearing surface [12]. In 1870, Stokes introduced an oval anterior flap instead of Gritti’s rectangular one [15]. The GSA allows for a sliding joint prosthesis, which more closely mimics the true knee joint than the AKA prosthesis’ artificial hinge joint. This improves the quality of life and independence for GSA amputees compared to AKA amputees [16].

### 3.1. 1900–1959

Our literature research did not identify any relevant studies on GSA from 1900 to 1959. This absence of studies may be attributed to the lack of popularity or adoption of the technique during this period.

### 3.2. 1960–1970

Martin and Wickham were the first to come up with a detailed report on their successes in employing the GSA technique in July 1962 [17]. They analyzed 80 amputations with a mandate in their own words: “A stump is valueless until it is useful”. Initially, they reported the reasons for the falling out of favor of GSA as the stump remaining after amputation is long, unfit for total end bearing, and not suitable for longer durations. They believe that if they could develop a light prosthetic replacement, the results would be better. Based on their analysis, there were five deaths in the immediate postoperative period; among the 75 surviving patients, 58 patients obtained wound healing by the first intention, and delayed healing was observed in 17 patients. A total of 5 patients underwent re-amputation due to severe infection among the group of delayed wound healing, while 44 patients were able to be fitted with a prosthetic limb. They concluded that GSA was the best option for PVD.

Middleton and Webster reported GSA results on 25 patients and noted that while GSA was recommended, the technique was poorly documented. In their study, there were two patients who died (8%). Among the 23 survivors, 18 (78%) had primary healing, 18 (78%) had a long follow-up, and 12 (52%) had prosthesis fitting. Six patients did not use prostheses due to a history of bilateral amputation, gangrene, dementia, or short follow-up. They concluded that their surgical outcomes surpassed Gilchrist’s, who reported a 27% mortality rate in 309 patients with above-knee amputations. They concluded that GSA was disliked because limb fitters were inefficient and that researchers should develop a lighter alternative [18,19].

Lishman reported on 37 patients who had a 6% perioperative mortality rate and an 80% primary healing rate. They agreed with Martin et al. and Middleton et al. that GSA is superior to AKA [20].

Martin et al. published their GSA results on 237 people in 1967. GSA outperformed other knee amputation methods, with a mortality rate of 5.9% and a 91% healing rate, with only 7.7% requiring re-amputation. They compared their findings to other BKA and knee disarticulation evidence. They reaffirmed the benefits of GSA, such as a high wound healing rate, quick recovery, low re-amputation, and low mortality rate. They also noted that only 123 patients (55%) were fitted with a prosthesis, mainly because of the mean age of 67 years with a history of cardiovascular disease. True functional outcomes evaluation was impossible in an aging and diseased population. Their results may have been different with younger patients [21].

In 1969, Weale criticized GSA in a supracondylar amputation with a patellectomy article [22]. Compared to BKA and through-knee amputation, GSA had 100% primary healing in 7 patients, while other techniques had lower primary healing percentages. He unexpectedly criticized GSA even though he compared the results with patellectomy, which was not the original technique of GSA. By contrast, the author used a different surgical technique, with symmetrical skin flaps, not U-shaped, as described for GSA. Their criticism was unjustified, lacked evidence, and was based on the authors’ own opinions.

During this period, most studies underscore the effectiveness of GSA, particularly in terms of high wound healing rates, quick recovery, and low mortality and re-amputation rates. Despite some criticism and challenges with prosthetic fitting, GSA was an effective option for PVD patients at the time.

### 3.3. 1970–1980

A published study by Newcombe et al. evaluated 41 patients who had undergone through-knee disarticulation. They criticized GSA and preferred through-knee amputation, claiming GSA was technically difficult to perform despite better primary healing and had inferior results than through-knee disarticulation regarding the rate of successful rehabilitation [23].

Doran et al. dispelled GSA’s need for a new prosthetic limb, its design, and its lightness. Supracondylar rather than transcondylar GSA was used, leaving a cross-sectional area roughly equivalent to the back of the patella. The sewn surface slopes upward and backward from the anterior aspect, preventing the patella from sliding forward. In their study, they evaluated 134 cases of PVD or ischemic limbs. They were satisfied with the results and reported that most of the disrepute ascribed to GSA is due to poor prosthetics and poor attachment of the patella, as many studies reported non-union of the patella in the stump as the reason behind the pain. Among the 106 survivors, there were 120 stumps and 83 primary healing (69%). Healing was delayed in 23 stumps (19%) but was eventually achieved, giving an overall cure rate of 88%. Fourteen cases of non-healing wounds required higher amputation levels (12%). They concluded that GSA is a good amputation method with low mortality, good healing, and a useful stump. They recommended it when the knee joint cannot be preserved [24].

Day HJ presented a review, “Stump and the Prosthesis”, describing the benefits of various popular Limb Salvage techniques. The author mentioned GSA for knee amputation. Despite its superior wound healing, the author did not recommend GSA due to prosthetic issues and the tendency for pain associated with the remaining patella [25].

Robinson summarized his 167 cases, including different types of amputations, and stated that GSA had limited use due to prosthetic and stump complications [26]. Interestingly, the author criticized GSA based on his personal opinion despite the absence of patients who underwent GSA in all presented cases.

During this period, some authors criticized GSA due to surgical technique difficulties, prosthetic fitting issues, and stump complications [23,25,26]. Despite these criticisms, one study of 134 patients demonstrated favorable outcomes for GSA in terms of good wound healing and low mortality rates [24].

### 3.4. 1980–1990

Beacock et al. studied 217 patients with a vascular disease whose 247 limbs were operated upon using GSA. They used a modified version of GSA and transected the femur at the supracondylar instead of the trans-condylar level, leaving a cross-sectional area roughly equivalent to that of the back of the patella. The angle of cut is such that the sewn surface slopes upward and backward from the anterior aspect to prevent the patella from slipping forward. In their paper, a 9.3 percent operative mortality rate was reported for 23 patients who died within 14 days of surgery. This left 224 stumps in the 194 survivors. The overall healing rate was 87%. Twenty (9%) patients underwent a subsequent mid-thigh amputation due to wound complications. Out of 194 patients, only 8 patients complained of stump tenderness (4%), but it was a major problem in only 3 patients (1.5%), as they could use their prosthesis for short periods. While patellar non-union does occur, it is usually asymptomatic. Of the 194 survivors, 112 (58%) used their limbs regularly. Of the total, 9.3% of patients returned to full employment. A total of 40 patients, of whom 18 were bilateral amputees, were confined to wheelchairs, but these patients had vascular disease. Therefore, a good functional outcome could not be predicted due to the patient population. Likewise, with better cosmetic designs, it may be acceptable to female patients with low patellar malfunction [27].

Campbell et al. performed a randomized prospective comparative study between GSA and through-knee amputation to evaluate the surgical outcomes. Twenty-four amputations were performed on 22 patients with PVD with a median age of 79 years. There were 11 patients in each group. A significant healing rate was observed in the GSA group when compared with through-knee amputation, with a lower risk of revision [28].

Houghton et al. conducted a detailed study to determine which of three amputations (through-knee, GSA, and AKA) offers patients the best chance of successful rehabilitation when BKA is not possible. The study was based on a questionnaire assessing postoperative rehabilitation. They analyzed 169 unilateral amputees using a self-devised scale, which scored various aspects such as the frequency of prosthesis use, type of assistive device needed, and feelings of instability while walking with the prosthesis. They found successful rehabilitation in 33% of above-knee, 62% of through-knee, and 44% of Gritti–Stokes patients. Both the GSA and through-knee amputation groups had significantly better rehabilitation outcomes than the AKA group. Although through-knee amputation showed better rehabilitation outcomes than GSA, the difference was not statistically significant. This observation could be due to a higher number of patients with PVD in the GSA group compared to the through-knee amputation group. Additionally, patients with through-knee amputation were significantly less dependent on a wheelchair than those with AKA. Despite the better results for through-knee amputation and GSA over AKA, the prosthetics were often considered cosmetically unappealing, especially by female patients. There was no difference between the three groups in terms of local stump complications or the reported frequency of prosthetic fitting. [29].

Studies from this period demonstrated favorable outcomes for GSA, particularly in terms of wound healing and better rehabilitation compared to AKA. Nonetheless, cosmetic concerns with prosthetics persisted, especially among female patients.

### 3.5. 1990–2000

Siriwardena et al. evaluated 598 patients who underwent BKA, GSA, and AKA using the walking ability index score. They concluded that GSA had a better walking ability index than AKA. However, they found that only 50% of GSA patients were satisfied with their prosthetic cosmesis, in comparison with 84% of AKA and 94% of BKA patients. Furthermore, prosthetic fitting took longer in GSA patients. However, these results should be interpreted with caution, as this study was published three decades ago, and current prosthetic evolution has improved prosthetic fitting and cosmesis [30].

Yusuf et al., in their comparative work between 117 AKAs, 144 GSAs, and 173 BKAs, dispelled the doubts regarding the procedure of choice for such conditions where BKA is not feasible in cases of arterial occluded limbs or vascular diseased limbs. After rehabilitation, they used Stanmore Mobility grades to evaluate all three techniques. GSA and BKA groups had better mobility scores than AKA, and there was no significant difference between the groups. Based on these findings, they strongly recommended GSA over AKA for affected limbs where BKA could not be performed [16].

Studies from this period share the same conclusion as previously published research, stating that cosmetic concerns with prosthetics remain the main challenge for GSA compared to AKA and BKA.

### 3.6. 2000–2010

Faber and Fielding used a novel method to confirm GSA usage. Amputations through the knee could have benefited from GSA, according to their experience and previous reports. They evaluated 66 limbs, 44 of which benefited from GSA [31].

We could not find other studies in this period after Faber and Fielding, as it seems that despite advances in prosthetic limb design, GSA usage declined in that period of time.

### 3.7. 2010–2023

Lim et al. were the first to advocate for GSA. They studied 65 closed-knee amputations in 58 adults aged 79 years over ten years. They reported a 78% overall healing rate. Six patients (9%) had amputation-related complications. They concluded their study by recommending GSA for limbs with occluded vascular disease [32].

Taylor et al. [33] hypothesized that GSA is superior to traditional knee disarticulation or transfemoral amputation and wondered why GSA is not used more frequently. They hypothesized that the patients treated with the GSA procedure would have a longer residual limb, improved wound-healing rates, an earlier time to prosthetic fitting, and a decreased revision rate as compared with the patients who had a standard transfemoral amputation. In addition, they also presumed patient-reported outcomes to be better in the GSA patient population. They performed the GSA as described previously by Doran [24], which they labeled in their subsequent work, detailing the surgical technique steps, which can be used as a noble reference for the technical note. The sickness impact profile (SIP) score of 15+ in 14 patients, including 7 females in GSA cases, was analyzed over four years. The study had limitations due to its retrospective nature, small sample size, and recall bias. They concluded by reiterating the advantages of GSA over through-knee amputation [33].

Jackson et al. chose GSA to be studied on 14 patients in a retrospective manner after reviewing 17 published articles regarding through-knee amputation. They attributed their success in ambulation to the latest prosthetic design with multicentric knee joints. They recommend GSA for all through-knee amputations [34]. Murakami and Murray conducted a meta-analysis of 17 published articles. GSA had the lowest risk of re-amputation. However, due to prosthetic design limitations, GSA may not be the best option for elderly vascular limbs. They also suggested more GSA research, given recent advances in limb prosthetics, in order to justify their use in patients with PVD. Aesthetic cosmetic prosthetic limbs are the most challenging part of GSA despite its advantages [35].

Theriot et al. conducted a retrospective study on 16 GSAs on 15 non-ambulatory patients over a year. They found that GSA had less blood loss, fewer transfusions, and fewer postoperative complications than AKA using other techniques. They advised GSA in all such cases, especially in non-ambulatory patients [36].

In a recent retrospective study by Brügger et al., the survival rate was examined 5 years after Gritti–Stokes amputation (GSA) and above-knee amputation (AKA) in individuals with vascular insufficiency. The study included 126 patients with an average age of 70 years, and a 37% survival rate was observed at 5 years. Prosthetic fitting and Gritti–Stokes amputation were found to be associated with better survival outcomes. The analysis revealed that prosthetic fitting was linked to improved survival, and in a multivariate analysis, Gritti–Stokes amputation was the only factor positively associated with prosthetic fitting. The study concluded that GSA is a valuable alternative to mid-thigh amputation in patients with PVD whenever possible to increase the chances of prosthesis fitting and, as a consequence, improve survival [37].

After reviewing the published data, orthopedic and vascular surgeons worldwide agree that when BKA is not an option due to vascular compromise, the GSA technique is the best option. With prosthetics fitting, early ambulation is possible. Table 1 summarizes our review findings.

## 4. Discussion

This review of published studies on GSA over the past 100 years found that most involved vascular patients with high comorbidity, which is not surprising, as these patients may lose some autonomy in the postoperative phase. Overall, GSA has demonstrated good outcomes, particularly in terms of good wound healing and low complication rates [16,19,20,21,24,27,32]. Compared to AKA, GSA has a lower risk of bleeding and transfusions, faster recovery, and lower mortality. Re-amputation is less common, and wound healing is faster than with BKA or through-knee amputations, resulting in better outcomes and fewer surgeries for these vulnerable patients.

GSA appears to have been criticized without supporting evidence, leading to its unfamiliarity, especially among young surgeons. Some authors criticized GSA without fully examining it [22,23,25,26,29], but this was over 40 years ago. Prosthetic fitting and cosmetic concerns may remain the main drawbacks of GSA. However, prosthetics have come a long way in the last few decades. With advancements in technology, prosthetics have become lighter and more aesthetically designed. Emerging techniques such as 3D-printed prosthetic limbs are now promising for improving prosthetic fitting for patients with GSA by providing better cosmetic appearance, personalized fitting, and affordability, even in developing countries [38,39].

Based on the above review, we believe GSA has the following advantages over AKA. We believe that the GSA shares many advantages with BKA and could be better than through-knee amputation and AKA.

### 4.1. Advantages of GSA over AKA

- Better wound healing due to the preservation of the superior geniculate artery [28,31].
- Minimal muscle dissection decreases blood loss and postoperative pain [19,21].
- Lower mortality rate [36].
- Reduced hospital stays due to rapid recovery [21,32].
- Early postoperative mobilization and rehabilitation and more rapid prosthetic fitting due to the limited degree of stump shrinkage [19,31].
- Lower energy consumption [40].
- Longer-lever arm amputation, which can help with transfers and sitting balance, is beneficial in bilateral amputees [33,41].
- Lower need for assistive devices for ambulation [41,42].
- Maintenance of the adductor insertions, which prevent unopposed abductors seen when not performing adductor myodesis [6].
- Maintenance of the quadriceps attachments to the patella, thereby resolving hamstring imbalance [36].
- Elimination of the need for an ischial-bearing prosthesis [19,41].
- The location of the AKA incision at the end of the stump makes it more susceptible to trauma, whereas the GSA incision is located on the posterior thigh, preventing this risk [36].
- Alignment of the AKA incision with the open medullary bone of the femur increases the risk of infection or wound dehiscence, whereas the GSA seals the medullary canal with the patella, reducing this risk [36].

### 4.2. Advantages of GSA over Through-Knee Amputation

- GSA provides longer flaps that are easier for wound closure [28].
- Reduced wound complications due to the absence of bulky femoral condyle [27,33,34].

### 4.3. Disadvantage of GSA over AKA

- When the patient is seated, knee joint lines are not at the same level [31,41].
- Longer time needed for prosthetic fitting [30,41].

## 5. Conclusions

This review aims to highlight and reintroduce GSA as a reliable technique. We reviewed the available evidence and discussed various aspects of GSA and its usefulness. Despite some criticisms regarding prosthetic fitting, most studies demonstrated favorable outcomes for GSA, particularly in terms of good wound healing. Furthermore, it provides faster recovery, better rehabilitation, and lower mortality rates compared to AKA. Prosthetic fitting and cosmetic concerns may remain the main drawbacks of GSA. However, with advancements in technology, prosthetics have become lighter and more aesthetically designed. Young surgeons should be aware of GSA and consider using it whenever BKA is not feasible before proceeding with AKA amputation.

## Figures and Tables

**Table 1:** Summary of Studies with the Conclusion as Favoring or Criticism about GSA.

Authors (Year)	No. of Patients with GSA	Mean Age in Years	% PVD	Surgical Outcome					Author’s Opinion Weather Favoring (GSA)/Criticize GSA
				Mortality Rate	Prosthetic Fitted	Overall Wound Healing Rate	Revision by a Higher Amputation	Complications	
Martin et al. (1962) [17]	80	69	100%	6.25%	58%	1° healing: 77% 2° healing: 23% Overall healing: 100%	6.25%	5 deep infections	GSA is a better option than AKA or knee disarticulation.
Middleton et al. (1962) [19]	25	61.1	96%	8%	30%	1° healing: 80% 2° healing: 100%	0	4 stumps tenderness 1 mobile patella 5 infections	GSA is superior to AKA and the best amputation that can be offered to atherosclerotic patients.
Lishman et al. (1965) [20]	37	NR	100%	6%	70%	1° healing: 81% 2° healing: 92%	8.1%	33% phantom pain	GSA is a better option than AKA.
Martin et al. (1967) [21]	237	67	100%	5.9%	55% (20/237 bilateral amputation)	1° healing: 78% 2° healing: 91%	7.7%	2 osteomyelitis 1 persistent ulcer 1 mobile patella	GSA is a better option. With low mortality, high wound healing rate, and a low re-amputation rate with useful stump. Bilateral GSA is better than bilateral AKA.
Weale et al. (1969) [22]	21	65	100%	0	100%	1° healing: 90% 2° healing: 100%	0	3 hematomas 1 chronic pain	Criticism of GSA. The author preferred supracondylar amputation with patellectomy rather than original GSA due to the risk of stump pain and mobility of the patella.
Doran et al. (1978) [24]	134	70	100%	11%	60% (15/134 bilateral amputation)	1° healing: 69% 2° healing: 88%	11%	6 stump tenderness (2 with major pain)	GSA is superior to AKA whenever BKA is not feasible.
Beacock et al. (1983) [27]	247	69	100%	9.3%	58% (18/247 bilateral amputation)	1° healing: 64% 2° healing: 87%	9%	8 stump tenderness (3 with major pain)	GSA is preferred to AKA or through-knee amputation.
Campbell and Morris (1987) [28]	12	79	100%	8%	25% (1 case with bilateral amputation, 2 hemiplegics)	1° healing: 75% 2° healing: 100%	0	1 infection 2 skin necrosis	Prospective randomized comparison study between GSA and through-knee amputation. GSA is superior to through-knee amputation regarding healing rate.
Houghton et al. (1989) [29]	27	64	69%	NR	52%	NR	NR	NR	Through-knee amputation has a better rehabilitation rate than GSA/AKA. Half of patients with GSA and through-knee amputation considered that prosthesis is unsightly.
Yusuf et al. (1997) [16]	144	76	100%	24%	30% (Stanmore mobility grade)	NR	6.25	NR	GSA has a similar mortality and rehabilitation rate compared to BKA, with a lower risk of re-amputation. GSA achieved better mobility than AKA. When a BKA is not possible, GSA offers better rehabilitation compared with AKA in patients with PVD.
Lim C et al. (2011) [32]	65	79	100%	24%	NR	1° healing: 70% 2° healing: 78%	1.5	1 hemorrhage 4 infections 1 skin necrosis	GSA is feasible for elderly patients and is associated with an acceptable mortality and overall healing rate. GSA is a good alternative to AKA and should be the standard amputation level.
Taylor et al. (2012) [33]	14	45	NA	NA	92% (35% without assistive device)	NR	7%	1 neuroma 1 infection	Study evaluated GSA outcomes in traumatic patients. GSA patients had significantly improved overall sickness impact profile (SIP) score in comparison with AKA.
Theriot et al. (2019) [36]	15	60	27%	0	0	1° healing: 73% 2° healing: 100%	0	3 stump infection 1 wound necrosis	Favored GSA recommends it to be performed in all possible circumstances.
Brügger et al.(2023) [37]	77	70	100%	63% at 5 years	NA	NA	NA	NA	GSA was the only factor positively associated with prosthetic fitting and by consequently improving survival.

PVD—peripheral vascular disease, GSA—Gritti–Stokes amputation, AKA—above-knee amputation, BKA—below-knee amputation, 1° healing—primary healing, 2° healing—secondary healing.

## Data Availability

The data presented in this study are available upon request from the corresponding author.
